# On the path to rabies elimination: The need for risk assessments to improve administration of post-exposure prophylaxis

**DOI:** 10.1016/j.vaccine.2018.11.066

**Published:** 2019-10-03

**Authors:** K. Rysava, M.E. Miranda, R. Zapatos, S. Lapiz, P. Rances, L.M. Miranda, M.C. Roces, J. Friar, S.E. Townsend, K. Hampson

**Affiliations:** a The Zeeman Institute for Systems Biology & Infectious Disease Epidemiology Research, School of Life Sciences, University of Warwick, Coventry, UK; b Institute of Biodiversity, Animal Health & Comparative Medicine, University of Glasgow, Glasgow, UK; c Field Epidemiology Training Program Alumni Foundation Inc. Quezon City, Philippines; d Global Alliance for Rabies Control Inc., Laguna, Philippines; e Provincial Health Office, Capitol Annex, Tagbilaran City, Philippines; f Office of the Provincial Veterinarian, Capitol Annex, Tagbilaran City, Philippines; g Asian Development Bank, Manila, Philippines; h Wise Monkey Foundation, Washington, USA

**Keywords:** Post-exposure vaccination, Dog-mediated rabies, Rabies prevention, Dose-sparing, Immunoglobulin, Intradermal, Intramuscular, Integrated Bite Case Management, IBCM, One Health, Risk assessment, Surveillance, Freedom from disease, Verification

## Abstract

**Background:**

Costs of rabies post-exposure prophylaxis (PEP) often remain high in regions where rabies has been controlled in dogs, presenting a challenge for sustaining rabies elimination programmes. We investigated the potential for bite patient risk assessments to improve PEP provision and surveillance in settings approaching elimination of dog-mediated rabies.

**Methods:**

We conducted a longitudinal study of patients presenting to animal bite treatment centres (ABTCs) on the island province of Bohol in the Philippines to investigate the health status of biting dogs and to quantify current expenditure on PEP.

**Results:**

Incidence of bite patients presenting to ABTCs was high (>300/100,000 persons/year) and increasing, resulting in substantial health provider costs. Over $142,000 was spent on PEP in 2013 for a population of 1.3 million. From follow up of 3820 bite patients we found that  >92% were bitten by healthy dogs (alive 14 days after the bite) and just 1.4% were bitten by probable or confirmed rabid dogs. The status of dogs that bit 6% of patients could not be determined. During the course of investigations of bites by suspect dogs, we were able to obtain samples for case confirmation, identify exposed persons who had not sought PEP as well as in-contact dogs at risk of developing rabies. We calculate that expenditure on PEP could at least be halved through more judicious approaches to provision of PEP, based on the histories of biting animals determined through risk assessments with bite patients.

**Conclusions:**

We conclude that a One Health approach to surveillance based on Integrated Bite Case Management could improve the sustainability and effectiveness of rabies elimination programmes while also improving patient care by identifying those genuinely in need of lifesaving PEP.

## Highlights

• Incidence of bite-injury patients and costs of PEP are high in Bohol Province, Philippines.
• Dog vaccination has controlled rabies so few patients (<2%) are bitten by rabid dogs.
• Risk assessments with bite patients can identify potential rabid dog bites.
• Investigations triggered by patient risk assessments enable early detection of rabies.
• This One health approach to surveillance could guide judicious PEP provision and improve PEP access.

## 1 Introduction

Timely rabies post-exposure prophylaxis (PEP) is required to prevent the fatal onset of disease in the event of a rabies exposure. If there is uncertainty about whether the biting animal could be rabid, PEP should always be provided; however, PEP is costly, not always available and difficult to reach for certain communities [1], [2]. An important premise of controlling and eliminating rabies at source through mass dog vaccination is that the public health sector will benefit through reduced rabies risk and therefore reduced expenditure on PEP [3]. This requires sensitive surveillance for decisions to be made about whether PEP is necessary, leading to appropriate and cost-effective PEP recommendations. Surveillance is, therefore, critical to guide practical decisions for both disease control programmes and public health actions [4]. When the ultimate goal is elimination, surveillance is particularly required to certify freedom from disease and to inform decisions regarding the cessation or scaling back of control programmes as well as budget allocation for treatment or prevention [5]. In the case of rabies, surveillance informs the need for continued mass dog vaccination and administration of PEP [6].

In spite of considerable progress in controlling dog-mediated rabies around the world, including much of Latin America [7], Eastern Europe [8] and parts of Southeast Asia [9], [10], [11], costs of PEP in these regions remain high. PEP demand often increases with the introduction of rabies control programmes, due to elevated awareness. Indiscriminate PEP administration can strain local and national healthcare budgets [12]. Eventually, PEP should become unnecessary (or only required for persons exposed elsewhere) if rabies no longer circulates locally, but methods to limit unnecessary PEP use have received limited attention. Laboratory surveillance is not necessarily satisfactory for decisions regarding PEP administration, given that animals are not always available for sampling, and diagnostic capacity is limited in some settings [13]. Moreover, delays to sample collection and laboratory diagnosis are commonplace, and PEP must be delivered promptly to be effective. Histories of biting animals provide valuable and oftentimes the only epidemiological information to inform PEP administration. Clinical information can in some instances provide a better measure of disease incidence than laboratory confirmed cases, depending on in-country capacity, infrastructure and resources [14].

Rabies control efforts are being implemented across the Philippines, with a number of islands and provinces on track for the elimination of both human and dog rabies [9], [11], [15], [16], [17]. However, international, national and subnational criteria to declare rabies freedom are not all aligned or fully accepted by practitioners, particularly in relation to how they may affect practical decisions about PEP administration. There is a need to strengthen surveillance to guide these policy decisions and public health practice. Operationalized surveillance criteria are needed to inform public health decisions and declare rabies freedom with confidence [18], for implementation across the Philippines and elsewhere in the world. We carried out a longitudinal study of animal bite patients on the island province of Bohol, in the Philippines, where an ongoing rabies elimination programme had controlled rabies [9]. Our aim was to identify potential criteria for informing PEP provision and guiding surveillance to verify freedom from disease and to evaluate PEP administration protocols to determine their potential economic and health benefits and risks. Using retrospectively collected data from anti-rabies clinics across the province, we explored prospective improvements for future surveillance strategies, providing valuable operational insights into their feasibility and potential utility in resource-limited settings.

## 2 Methodology

**Fig. 1:**
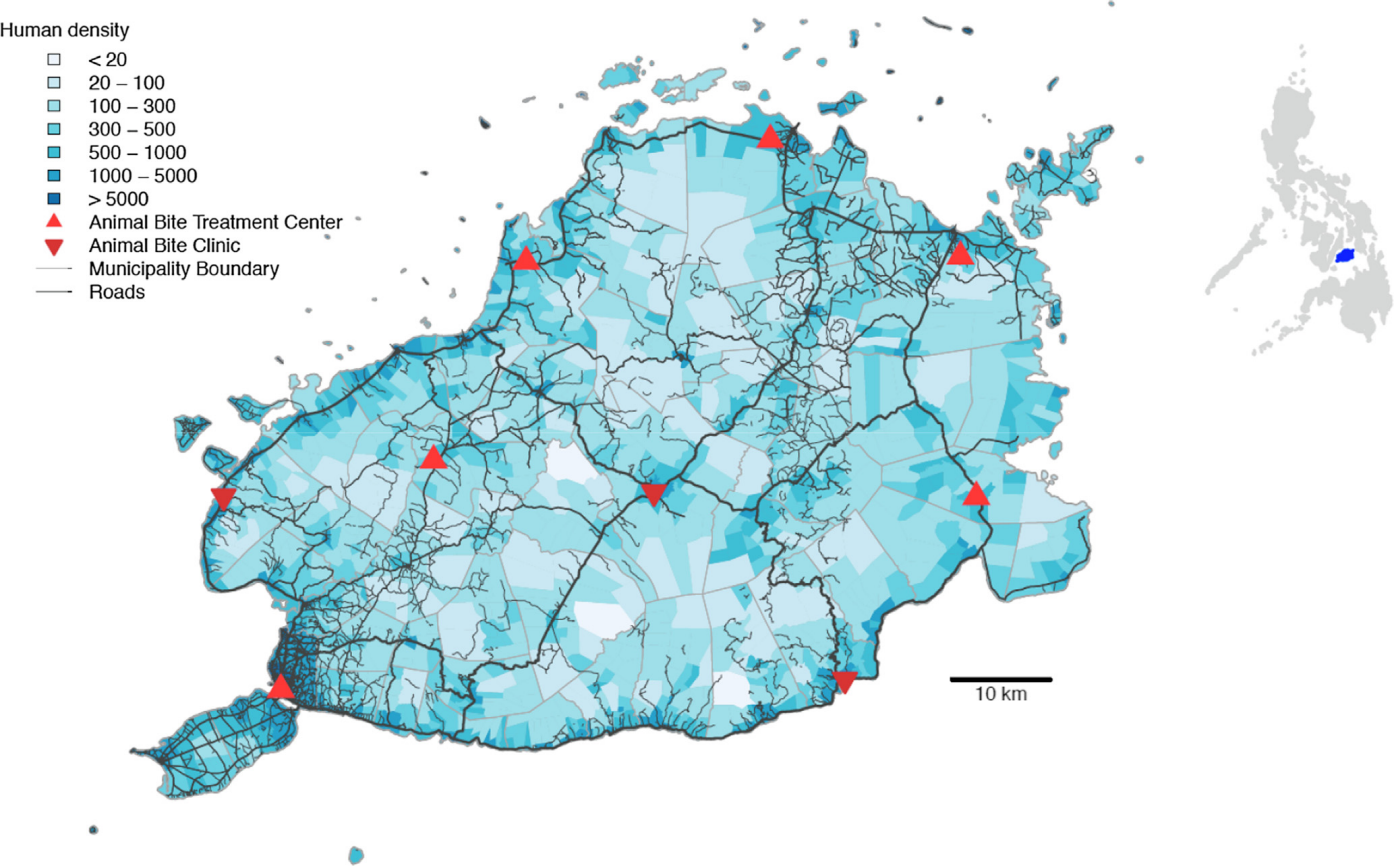
Bohol Province in the Central Visayas, Region VII of the Philippines, showing the human population density and locations of clinics providing PEP to bite patients. Human density was estimated at the barangay-level (village) based on the 2015 census. The inset shows the location of Bohol (blue) in the Philippines.

*Study site:* We established a longitudinal study of dog bite-injury patients during 2013 (from January to December) on the island province of Bohol, in the Central Visayas (Region VII). The province comprises 48 municipalities and had a population of 1,313,560 in 2015 (Fig. 1) [19]. A rabies control programme in Bohol was established in 2007, involving annual vaccination of domestic dogs and promotion of responsible dog ownership [9].

*PEP provision:* At the time of the study, the first two doses of human rabies vaccine and rabies immunoglobulin (RIG) were provided free-of-charge from government-run Animal Bite Treatment Centres (ABTCs) located within hospitals in Bohol and could be bought privately from Animal Bite Clinics (ABCs, sometimes referred to as private Family Vaccine and Speciality Clinics, FVSCs). ABTCs administer vaccine intradermally (ID) following the updated Thai Red Cross regimen (TRC) with two 0.1 mL doses (to deltoids) delivered on day 0, 3, 7 and 28. Five private hospitals within Tagbilaran City and in the city outskirts used the intramuscular (IM) route for PEP administration. The ABTCs and ABCs (as part of a Memorandum of Agreement with the provincial government) are required to report quarterly to the Provincial Health Office (PHO) supplying data on bite incidence for the provincial rabies control programme, whereas private hospitals do not have a mandate to report bite patients. Across the province, 52 Rural Health Units (RHUs) operate where initial first aid is provided to bite victims together with tetanus toxoid vaccination. RHUs refer bite victims for PEP; ABTCs generally do not provide PEP without a referral from an RHU. The Research Institute for Tropical Medicine of the Department of Health in Metro Manila trains staff from the ABTCs in rabies prevention and administration of PEP [20]. ABCs have their own training which is not accredited by the Department of Health.

*Bite patient follow up:* During this longitudinal study all ABTCs and ABCs were visited monthly throughout 2013 and records were collected on all animal bite patients and PEP use. The doses of vaccine and RIG administered to patients were recorded, but no changes to routine PEP provisioning were made during this study. At the start of the study clinicians were requested to record bite patient phone numbers within their standardised animal bite registry books (S1) on their first consultation (day 0) to enable phone call follow up. Bite victims were advised to observe the biting animal and immediately call the clinic in the event of the animal showing any behavioural and/or health-related changes. Nurses at the ABTCs were instructed to call patients (or parents/guardians in the case of minors) fourteen days after their first clinic attendance to complete a short questionnaire over the phone to identify whether the incident involved a suspect rabid animal (S2). Specifically, any biting animal reported to be sick, to have died, or have been killed or be untraceable during the 14 days following the bite incident, was considered suspect and triggered an immediate field investigation (S3). Under routine government surveillance, field investigations are only conducted in the event of a human death [20]. The rabies nurse coordinator (RZ) assisted with the phone call follow up during monthly visits to the ABTCs because of the high volume of bite patients. Staff at RHUs were also requested to report to the rabies nurse coordinator immediately after receiving information on any suspicious case/ series of suspicious events to facilitate rapid investigation.

We used the following case definitions to classify the status of biting dogs. We considered a biting animal to be *probable rabid* if it exhibited unusual aggressive or lethargic behaviour and either died, was killed or disappeared during the 14 days following the bite. Unusual aggressive behaviour included biting multiple people and/or animals and other clinical signs of rabies as per WHO case definitions [21]. Bite incidents reportedly caused by dogs vaccinated within the last 12 months or dogs showing no clinical symptoms of rabies during the 14-day observation period were classified as *non-cases*.

Investigations involved visits by staff from the Office of the Provincial Veterinarian (OPV) and the PHO, typically a veterinarian and a rabies nurse, to the patient’s village (barangay). The history of probable rabid animals were traced via interviews with the bitten person, the animal owner and witnesses of the incident to find other animals and people that were potentially exposed. Probable rabid animals were euthanized, as were identified unvaccinated in-contact animals. Regardless of the category of bite, a dog vaccination history of 2 consecutive years as evidenced by an owner’s vaccination card precluded euthanasia, and the animal was subjected to the required observation period. Exposed persons were interviewed to find out what procedures had been taken after the bite. If they had not sought medical advice they were advised to go initially to the RHU for subsequent referral for PEP. If a sample was obtained from an animal, the animal head would be sent to the Diagnostic Laboratory in Cebu for confirmatory testing using the Fluorescent Antibody Test (FAT) after the direct rapid immunohistochemical test (dRIT) at the Bohol OPV indicated positive.

**Fig. 2:**
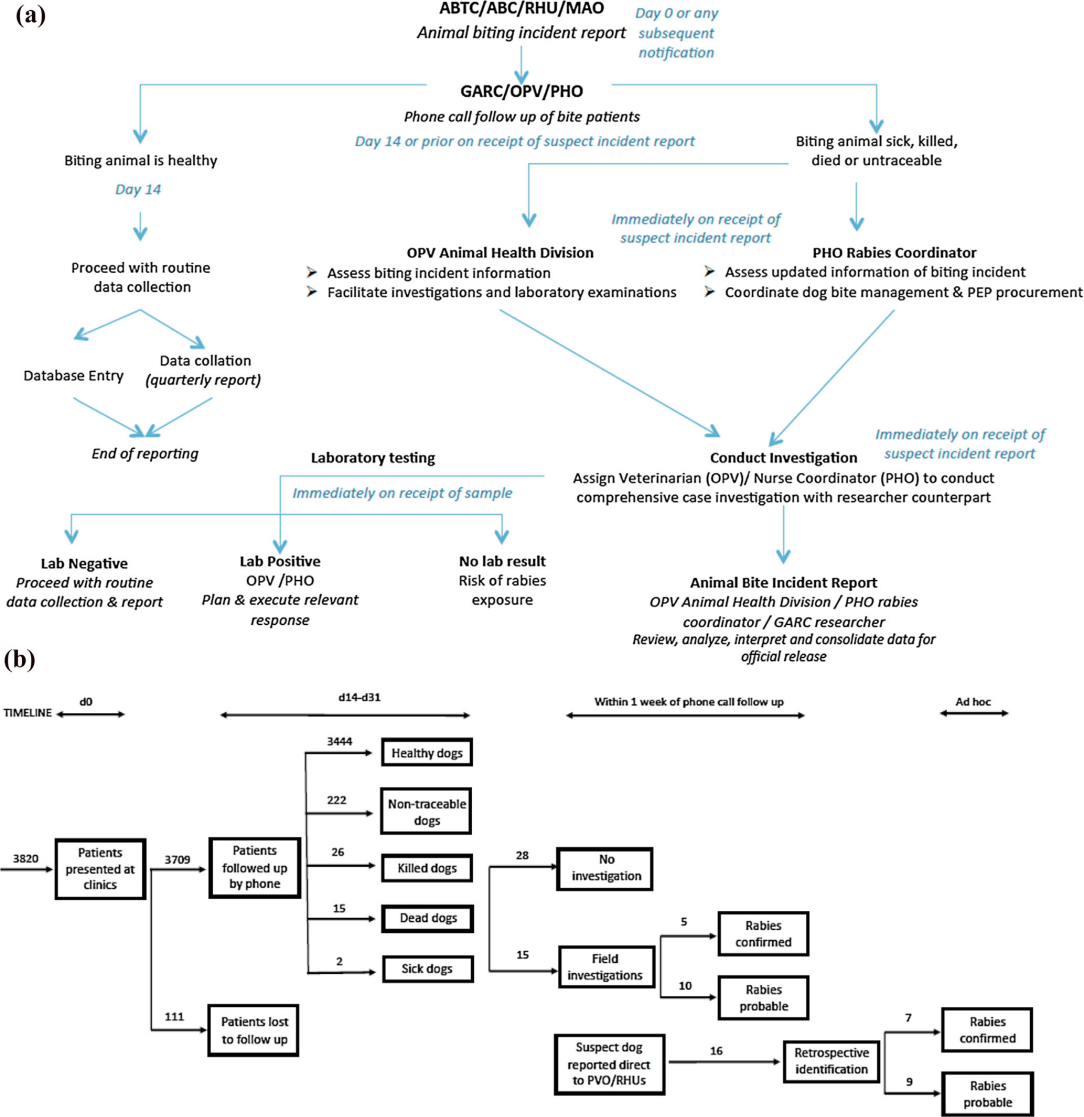
Study protocol indicating process (A) from recording of bite victim to completion of investigation and (B) outcomes of study. Bite incidents are recorded by health workers when patients report to anti-rabies clinics. Health Workers aimed to call patients 14 days after their first clinic presentation to ascertain any changes in the biting animal's behaviour or health condition (in practice calls were made at least within 30 days of the patient presentation). In the instance of the biting animal being sick, found dead, killed or untraceable, field investigations were conducted, followed by brain sample collection where relevant and available. Under the protocols operating in the Philippines at the time of the study, PEP was continued irrespective of field investigation or the status of the biting animal. ABTC = Animal Bite Treatment Centre, ABC = Animal Bite Clinic, RHU = Rural Health Unit, MAO = Municipal Agricultural Office, GARC = Global Alliance for Rabies Control office, OPV = Office of the Provincial Veterinarian, PHO = Provincial Health Office.

Clinic registry data were entered and submitted to a tailor-made surveillance software used for research purposes (www.wise-monkey.org), together with the subsequent follow up information. The process of data collection, criteria for follow up and procedures for coordination of investigations with the OPV and PHO are outlined in Fig. 2 and investigation forms (S1 – Primary patient data, S2 – Patient phone follow up, S3 – Animal Bite Investigation) are provided in the Supplementary Material. Aggregate records of bite patients and human rabies cases since 2007 were obtained from the PHO.

*Analysis:* We used the clinic registry data and the bite patient follow up forms (S1-3) to categorize the proportion of patients exposed by confirmed and probable rabid dogs, and those classified as non-cases. We collated costs of PEP provision as well as the costs needed for an enhanced surveillance approach using Integrated Bite Case Management (IBCM) that would cover the required field investigations. We then calculated the potential economic benefits of the following three scenarios: (1) current practice of indiscriminate PEP administration without bite patient investigations; (2) enhanced surveillance with provision of the first dose of vaccine only (no RIG) to patients bitten by apparently healthy animals on the basis of the health workers’ risk assessment (no signs of illness described), with further doses withheld following confirmation that by d3 the animal remained healthy and (3) as per scenario 2 but with no PEP for patients bitten by dogs vaccinated within the last 12 months (a less risk averse and potentially more cost-saving approach). Information collected during patient follow up and animal bite investigations was used to calculate the number and proportion of probable and confirmed dog rabies cases, vaccinated dogs and patients that would have been administered RIG and vaccine on day 0, 3, 7 and 28 in 2013 according to these three scenarios. Code and data for reproducing the figures are available at: https://github.com/katiehampson1978/rabies_risk_Bohol.

## 3 Results

*PEP provision:* At the start of the study PEP was available from eight centres in Bohol: five ABTCs and three ABCs (Fig. 1). However, in October 2013, partway through the study, Bohol was struck by a major earthquake (moment magnitude scale of 7.2), which destroyed infrastructure across much of the island. At least 2 ABTCs were completely destroyed, and for the final 3 months of the study until the beginning of 2014 bite patients were instead referred to the ABTC in the provincial capital. Following the earthquake one ABTC (Catigbian District Hospital) opened that aimed to provide better services for citizens within the interior district of Bohol and two ABCs in Inabanga and in Catigbian municipalities were also opened, but were not sustained because they co-occur in hospitals with ABTCs. One ABC in Loon was closed following the earthquake.

On Bohol, ABTCs use a strategy aiming to increase compliance, by providing vaccine doses free-of-charge on day 0 and day 3, with patients expected to pay for doses on day 7 and day 28. Two vaccines were used: Rabipur, a purified chick embryo vaccine (PCEC) supplied in 1 mL vials and Verorab, a purified verorab vaccine (PVRV) supplied in 0.5 mL vials. In 2013 these vaccines were sold in pharmacies at 1600 pesos and 1300 pesos (equivalent to $36.0 and $29.3 USD) per vial, for Rabipur and Verorab respectively. On a patient’s first clinic visit PEP is prescribed by a doctor or nurse according to the vaccine available at that clinic. Patients using Rabipur and returning for their 2nd or 4th dose wait for another bite victim to attend the clinic to share the costs of the vial. Patients prescribed Verorab go straight to the pharmacy to buy a vial, to be administered at the ABTC.

Privately run ABCs charged bite patients 700 pesos for each clinic visit where patients received two ID vaccinations of Abhayrab vaccine (PVRV), which costs 800 pesos per 0.5 mL vial. Human RIG (HRIG) was only available in one pharmacy in the provincial capital, Tagbilaran city, at 5000 pesos per 10 mL vial ($90). Equine RIG (ERIG) was widely available and sold at 1200 pesos per 10 mL vial ($22). ABCs typically provide RIG for all category III exposures, using Vinrab, an ERIG. The dose used depends on the weight of the patient, with approximately 1 vial used per 10 kg, with most bite patients requiring between 3 and 6 vials depending on their size and weight. The Memorandum of Agreement with the provincial government also required that ABCs give the last dose of vaccine for free, but this is not often practiced.

The protocol for PEP administration is that patients with category II exposures (superficial bites without any signs of bleeding, from the neck downwards) should observe the dog and if alive after 14 days should discontinue PEP without completing the 4th scheduled vaccinations. Category III exposures (bites from the neck upwards or multiple bites on any parts of the body that were bleeding) are advised to always complete the 4 doses plus immunoglobulin (RIG), even if the dog remains healthy. ABTCs rarely record the vaccination status of the dog.

### 3.1 Bite patient incidence

During the 12-month period of the study we compiled individual records of 3820 patients bitten by dogs who received PEP from the ABTCs and ABCs in Bohol, shown in Fig. 1. Paper records from ABTCs and the OPV were destroyed during the earthquake, but efforts were made to continuously collect data and enter records into the online database. A total of 5457 bite patients were recorded in the Provincial Health Office report in 2013, including 515 bitten by cats and 34 by other species (species not recorded). We were unable to fully determine the mismatch between the individual records and aggregated report (3820 versus 4908 dog bites), as data were not grouped by clinic or month (and may have involved double counting), but there was also loss of individual records and difficulties in successfully calling patients following the earthquake. During the first month of the study (January 2013), training of nurses was underway, therefore phone call follow up was also not completed for a proportion of patients.

**Fig. 3:**
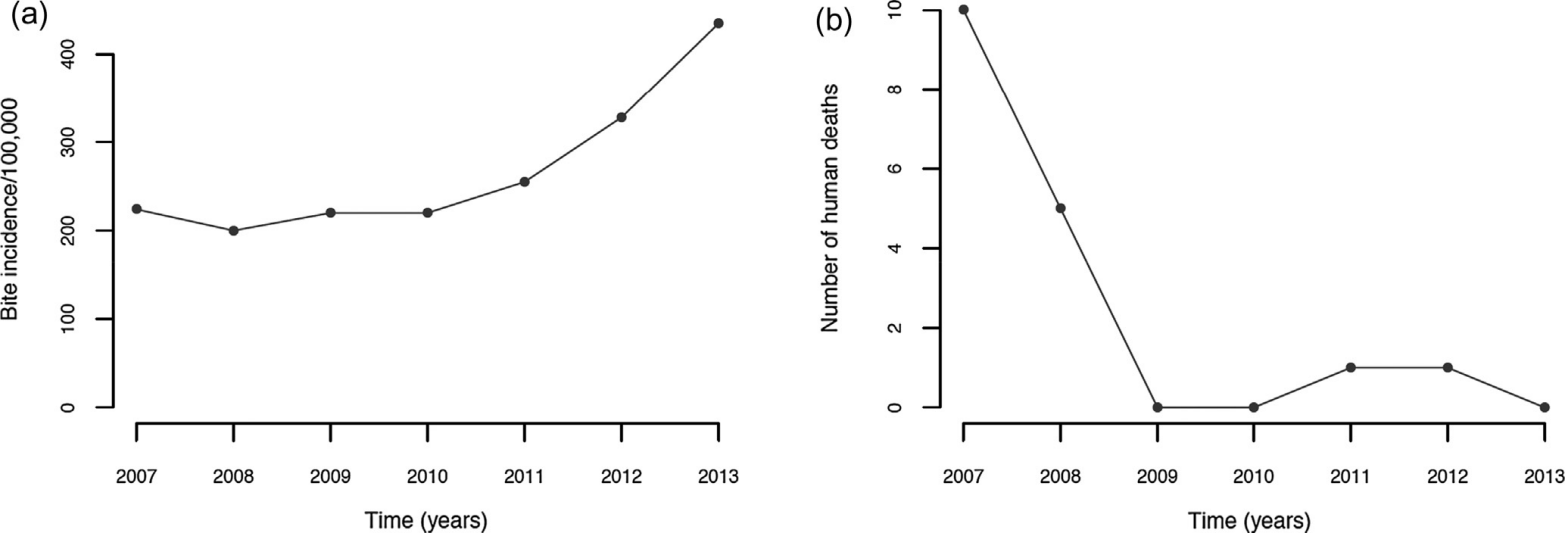
Annual time series of (A) bite patient incidence reporting for PEP per 100,000 and (B) human rabies cases from the beginning of rabies control programme on Bohol in 2007 until 2013.

**Fig. 4:**
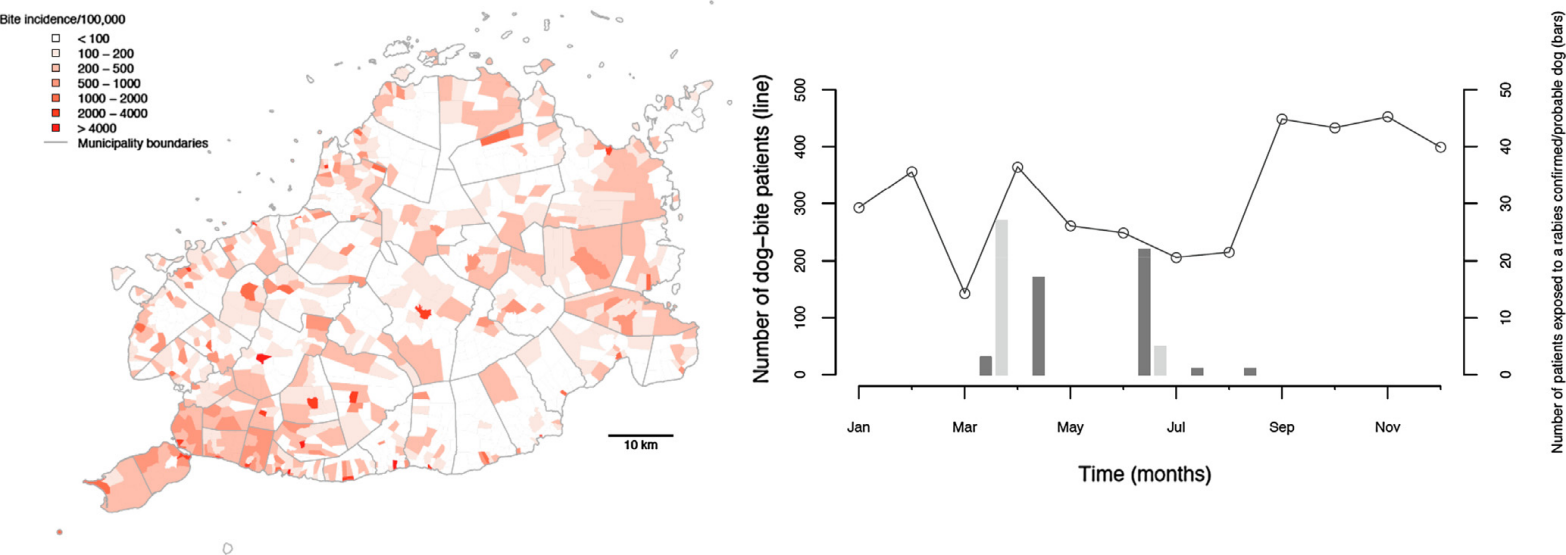
The distribution and monthly time series of dog bite patients and confirmed/probable rabies exposures receiving PEP from ABTCs and ABCs in 2013. Dog bites are shown by the line and rabies exposures are shown by the bars (bites and meat consumption shown in dark and light grey, respectively).

These data corresponded to a high annual incidence of 435 bite patients per 100,000 persons (or 304 dog bite patients per 100,000 persons from the compiled patient forms). Reports of bite patient incidence increased from 2007, when the Bohol rabies control programme was set up, until the end of 2013 (Fig. 3), with an average of 268 patients per 100,000 persons per year (95% confidence Intervals (CI): 190–346). Reported bite patient incidence also varied geographically, with an average of 213 bite patients (CI: 185–241) per 100,000 per year at the barangay level, but many barangays (>40%, 470/1109) had zero bite patients in 2013, and 36 (3.2%) had an incidence exceeding 1000 bite patients per 100,000 per year (Fig. 4).

**Fig. 5:**
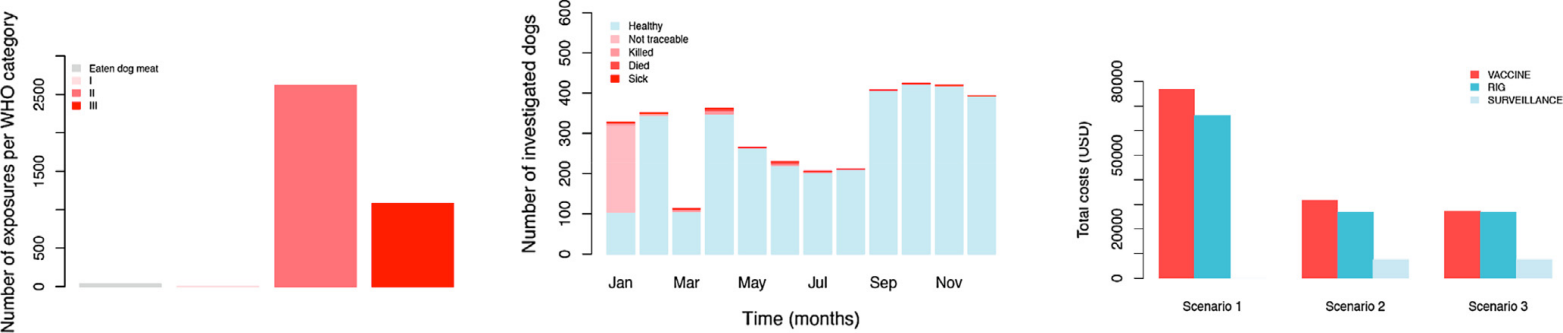
Breakdown of patients attending ABTC/ABCs in 2013 according to their exposure status and implications for PEP expenditure. (A) Number of patients classified per WHO exposure category; (B) monthly time series of bite patients according to the status of biting animals after the 14-day observation period classified from phone call follow up and (C) estimated PEP and surveillance costs under the current and two alternative scenarios. Training of nurses in phone call follow up of patients was only completed in January 2013. Hence not all patients were followed up (phone numbers were not previously recorded routinely) in January 2013. A total of 15 field investigations were initiated following identification of suspect dogs during phone call follow up.

Most patients attended government-run ABTCs with only 11% of bite victims attending private clinics. Throughput of ABTCs varied considerably, with the main ABTC in Tagbilaran receiving around 22 patients per day (maximum of 86 and median of 16), including both new patients and those returning for subsequent PEP doses. In smaller peripheral ABTCs, the throughput of patients (new and returning) was on average 5 per day. The majority of patients had category II bites (70%, Fig. 5A). The individual bite patient records indicated that all patients received one or more PEP doses, with 72% and 67% receiving 2 and 3 doses respectively and just 32.5% receiving the fourth and final dose (day 28).

**Fig. 6:**
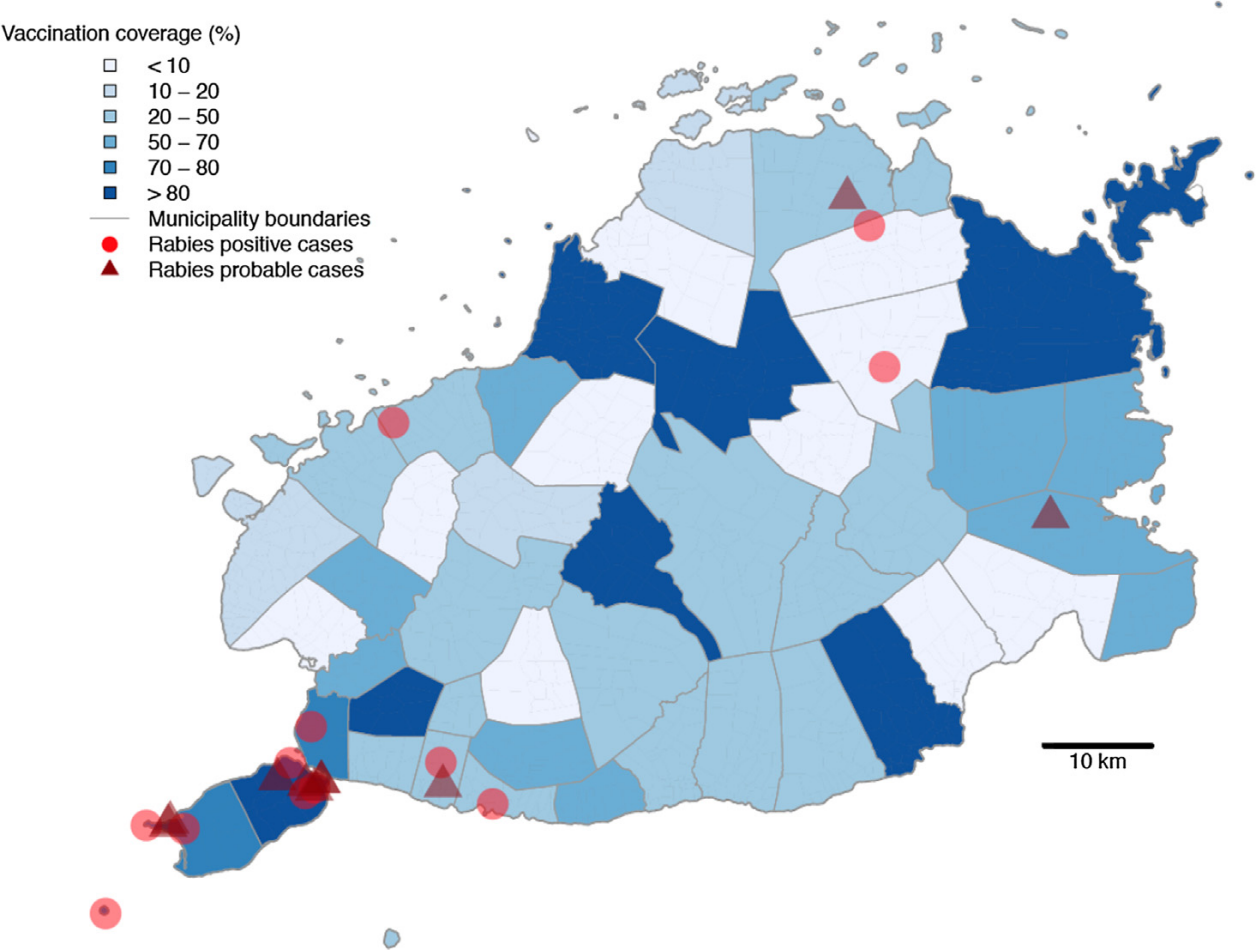
Locations of confirmed and probable rabid animals identified during investigations. The estimated dog vaccinated coverage at the municipality level based on dog vaccinations in October 2013 is also shown.

On the basis of phone call follow up with over 75% of bite patients recorded in the primary animal bite registers in 2013, we conducted 15 field investigations. Laboratory diagnosis confirmed rabies in 5 biting animals from these investigations (10 animals were unfit for testing - but were considered probable rabies on the basis of clinical signs). Seven more dog rabies cases were identified and confirmed retrospectively (carcasses found), but due to delays in reporting no field investigations were instigated following these incidents. We found that in total only 44 people were exposed by confirmed rabid dogs (n = 10) or probable rabid dogs (n = 19). In addition to the 44 persons directly exposed to rabies, another thirty-two consumed the meat of two confirmed rabid dogs (well cooked n = 18, partially cooked n = 9, rare n = 5); (Fig. 5A,B). The locations of the confirmed and probable rabid dogs are shown in Fig. 6, in relation to municipal-level dog vaccination coverage. All cases were tested using dRIT at the Bohol OPV and confirmed by FAT at the regional laboratory, Cebu, with both tests in full agreement. A further 222 (5%) patients were bitten by dogs that could not be traced (Fig. 2, Fig. 3, Fig. 4); however, most of these were from January 2013 when training was underway and phone numbers had not been collected to enable follow up. All other patients interviewed were bitten by dogs that remained alive and healthy for 14 days after the bite, of which 18% were dogs reported to have been vaccinated in the last 12 months.

On investigation of the confirmed/probable biting animals, other people and animals were identified that were bitten. Nine dogs that were bitten by these probable rabid dogs were euthanized during the course of investigations, even though some had previously been vaccinated. One of the people bitten in March 2013 did not seek PEP due to financial difficulties, but the PHO provided PEP to the victim when the dog was confirmed rabid. Investigation of a dog that bit four school children in June 2013 led to the identification of another bitten child who was then directed for PEP. No human rabies cases were recorded in 2013, but during investigations one person (female, 14 years old) was identified who was bitten on the foot by an unknown dog prior to the 2013 earthquake in the same municipality where the suspect rabid dog bit 5 children in June 2013. She did not receive any PEP and did not clean or wash the wound. In January 2014 she started to show symptoms consistent with rabies (vomiting, headache, restlessness, hypersalivation and hydrophobia) and died shortly after admission to hospital. However, her cause of death was not confirmed (laboratory confirmation of human rabies is not routinely carried out in the Philippines).

From the individual records of bite patients we calculated that at least $142,600 USD was spent on PEP in 2013, $76,650 on vaccine and $65,950 on RIG, assuming use of Rabipur with vial sharing, a wastage factor of 25% and an average of 4.5 ERIG vials per person. Since patients pay for the 3rd and 4th dose, the cost to the health provider for vaccine was estimated to be $48,912. However, given that the vast majority of biting animals were healthy (95%), more judicious use of PEP could have resulted in substantial savings. Additional costs would be required to strengthen surveillance activities to include bite patient follow up and coordination with the OPV and PHO. We estimated that under enhanced surveillance in Bohol (scenarios 2 and 3) monthly running costs would have been around $640 ($7680 USD annually), and that expenditure on PEP could have been reduced by more than half. Assuming that all patients bitten by healthy animals had been given only 1 dose of vaccine and no RIG (scenario 2), with all other patients completing 3 doses, the total expenditure could have been minimized to $66,226 USD. Whereas if no PEP had been administered to patients bitten by dogs vaccinated in the last 12 months (scenario 3) total expenditure would have been reduced to $61,581 USD (Fig. 5C).

## 4 Discussion

We found a high and increasing incidence of bite patients reporting to ABTCs/ABCs on Bohol, exceeding 300/100,000 persons per year, even though the incidence of dog rabies was very low. These data show the substantial cost to the government of indiscriminate PEP provision, despite the effectiveness of the provincial rabies control programme in dramatically reducing rabies incidence. Phone call follow up of bite patients shows that suspect rabies cases that require investigation can be identified from simple criteria. Unvaccinated biting animals with unusual aggressive or lethargic behaviour should be considered high risk with investigation needed to confirm their clinical status. If within days of biting an animal dies, disappears or is killed with signs of rabies, PEP should be completed by bite victims. Investigations triggered by suspicious bites can also further identify other persons exposed to rabies who have not sought care as well as in-contact animals at risk of rabies. This approach has potential to guide judicious PEP provision and could pay for itself through reductions in costly PEP use, whilst strengthening surveillance. However, implementation research is urgently needed to operationalize enhanced surveillance and judicious PEP provisioning and generate lessons for best practice.

High incidence of dog bites and PEP demand is a concern in endgame settings when budget pressures may compromise dog rabies control efforts at this critical juncture [22]. More generally, indiscriminate PEP administration is expensive and can lead to vaccine shortages. In the Philippines PEP use has increased annually, with the Department of Health reporting a rise from 783,663 animal bites in 2015 to 1,362,998 in 2016 [12]. Growth in PEP demand at this rate is unsustainable. One Chief of Hospital explained “*We urgently need to rationalize PEP…. We cannot afford to keep vaccinating patients* … *we vaccinate so many that we run out and then have to send people away and ask the mayor for more vaccines*”. In response to limited PEP availability in 2018, emergency measures were instated, with PEP only administered to patients with category III exposures and category I and II exposures advised to purchase vaccines from private clinics. Non-prequalified vaccines were used where WHO recommended vaccines were unavailable and in some provinces dog vaccination effort increased. The urgent need for discerning PEP protocols is clear; identifying high risk exposures can prevent unnecessary risks when vaccine stocks are limited. Our analyses suggest that an Integrated Bite Case Management (IBCM) approach to surveillance is a highly cost-effective strategy that can inform judicious PEP provisioning, and reduce PEP costs by more than half, with additional savings possible through implementation of the latest WHO recommendations for PEP [6].

Risk assessments guide PEP administration in countries that are free from dog rabies [23], [24], and similar methods have recently been applied successfully in rabies endemic settings [25], [26], [27]. We assessed the risk of infection retrospectively and no changes to PEP provisioning were implemented in our study, but we highlight the potential public health and economic benefits a risk-based approach to rabies surveillance might have in the Philippines, as do studies from more endemic settings [28]. IBCM has been shown to be feasible and cost-effective in highly endemic settings and to substantially improved patient care, averting a number of rabies cases that would have otherwise likely occurred [25], [29]. Such an approach relies on the sensitivity of clinical identification of rabid dogs, which is very high [30], [31], while ensuing investigations enable timely containment and removal of exposed and/or infectious dogs. We suggest that in the Philippines, where most dogs are owned and dog vaccination programmes are in place, initial risk assessments at ABTCs, ABCs and RHUs could identify suspect bites from unvaccinated dogs showing suspicious behaviour such as lethargy or unusual aggressiveness. In these settings approaching elimination relatively few investigations would be required, making timely investigations feasible and keeping costs low (Fig. 5C), though in more endemic settings more consideration would need to be given to costs and capacity. This approach would enable health workers to advise patients on day 3 to complete PEP for all bites by probable rabid animals (including those that cannot be traced), but discontinue PEP for animals that remain healthy. Intersectoral collaboration and effective communication between the health and veterinary sector would be critical to the success of such an approach [32], with frontline health and veterinary workers requiring training and support to implement these new procedures and make confident decisions. Given the financial strain of high PEP use in the Philippines, such an approach would be warranted.

In Bohol, the incidence of patients with bite injuries is very high, but comparable to other parts of Southeast Asia [33], [34], [35], [36], in part due to sensitization efforts and improved PEP access. As a result, dog bite numbers do not reflect rabies incidence in humans or dogs. Yet, even with this improved PEP access through free provision and decentralized ABTCs, occasional human deaths occur, highlighting how perceptions of risk can differ and how difficult it is to change risky behaviours, such as visiting traditional healers, and slaughter and consumption of dog meat [37]. By way of example, investigations in 2014 revealed that of three people bitten by a rabid dog on Bohol in October 2014, none sought PEP and the dog meat was consumed by several people. The dog owner assisted one of the bite victims to a local traditional healer who performed “tayhop” and other rituals for treatment of the dog bite. This bite victim died of probable rabies in November 2014 (after which the others were vaccinated). The MAO had conducted dog vaccination in October 2014 and shown a sensitization film which the bite victim watched, yet still did not seek PEP. Shortly after her death a dog head sample was collected from the same municipality and tested positive for rabies by FAT. The frequent dog meat consumption that we found also poses risks if associated with human-mediated movement of dogs for trade, which could lead to incursions and has been reported in other Asian countries [38].

The situation on Bohol highlights challenges to achieving and sustaining rabies elimination. The small number of rabies cases detected in 2013 were likely the result of residual focal transmission or may have included incursions from other islands. Incursions are an obstacle to maintaining rabies freedom [39], [40] and have been shown to pose a threat to elimination goals in the Philippines, despite being an island archipelago [41]. In practice, when incursions are detected, stakeholders may consider control efforts to have failed and be reluctant to report such instances. But incursions are to be expected when rabies is circulating in neighbouring areas [10], [42], [43] and sensitive surveillance is critical for their early detection and containment [44]. Current policy in the Philippines is to only conduct investigations of probable human rabies cases and of confirmed dog rabies cases [20], but the integrated approach that we advocate - using bite patients as sentinels for rabid dogs - has potential to substantially increase case detection [45] and could generate critical data for verifying freedom from disease [18].

Our aim was to follow up every bite patient to investigate whether biting animals were rabid or healthy. However the 2013 earthquake destroyed some ABTCs and limited our ability to recover all records and identify all bite patients, leading to discrepancies between aggregate bite data and individual record numbers. Nonetheless, we interviewed the vast majority of patients and were able to classify most bites according to risk. We struggled to trace all bite victims during the first month of the implementation of the study when PHO staff at ABTCs began to implement the new protocols, but thereafter were able to very effectively identify and classify the status of animal bites (Fig. 5B). However, investigations were not completed for all suspect biting dogs, highlighting the urgent need to strengthen channels of communication and vigilance among health and veterinary workers essential for effective surveillance during the endgame. A limitation of our study were the assumptions required for calculating PEP costs. We used relatively conservative simplifications to account for the variety of vaccines used, their prices and vial sizes. We therefore expect that both the costs of PEP and potential savings that could be made through more judicious administration likely exceed what we report, especially if dog vaccination status is better reported as part of IBCM. However, we also recognize that operationalizing procedures for more judicious PEP administration is likely to be difficult to put into routine practice and will require implementation research. Nonetheless, examples of effective implementation and benefits of IBCM in high incidence settings suggest considerable promise [25], [29], and this approach is recommended by WHO [21].

## 5 Conclusion

Our aim was to determine epidemiological surveillance procedures and quantify economic benefits of appropriate judicious PEP administration in endgame settings aiming to eliminate dog-mediated rabies. We conclude that risk assessments of bite patients at anti-rabies clinics would provide pertinent information to trigger investigations that are a sensitive means to detect rabies cases. Such a One Health approach could significantly improve the administration of expensive but life-saving rabies post-exposure vaccines, including identifying rabies exposed persons who would otherwise not receive PEP. This approach has been shown to be cost-effective in highly endemic settings [29], and implementation studies in other settings such as the Philippines could generate valuable lessons for best practice and transferability to other contexts. There is an ethical imperative to improve access to PEP in endemic countries where human rabies deaths still occur, but escalating costs also need to be curtailed and surveillance strengthened for elimination programmes to successfully reach their target. Our longitudinal study suggests an integrated One Health strategy to reduce unnecessary PEP use, while at the same time strengthening surveillance, improving sustainability of rabies control programmes and identifying high-risk exposures that may not otherwise seek care.

## Conflict of Interest Statement

None.
