# Supplementary material for: On the path to rabies elimination: The need for risk assessments to improve administration of post-exposure prophylaxis
Source: Vaccine. 2019 Oct 3;37(Suppl 1):A64–72. doi: 10.1016/j.vaccine.2018.11.066 (PMC6863041; doi:10.1016/j.vaccine.2018.11.066)
Supplement: Supplementary Data 1 [file mmc1.pdf]

FORM 1. Primary patient data upon visit of patient to clinic

Clinic: (Candijay/Carmen/Jagna/Loon/Tagbilaran/Talibon/Ubay)

VICTIM PROFILE

**Name**	______________________	
**Age**	____ years<br>____ months	
**Sex**	☐ Male ☐ Female	
**Status of the victim**	☐ Alive ☐ Dead, date of death(MM/DD/YYYY) _____________	
**Address of the victim**		
House name/number and street	______________________	
Sitio	______________________	
Barangay	______________________	
Municipality/city	______________________	
**Telephone Number**	______________________	*VITAL INFORMATION*
**Bite date (MM/DD/YYYY)**	______________________	*VITAL INFORMATION*
**Date Type**	☐ Date bitten ☐ Date of first treatment	
**Acitivity Date Estimate**	☐ Accurate ☐ +/- 1 day ☐ +/-1 week<br>☐ +/- 1 month ☐ +/- 1 year	
**Location where biting incident happened**	☐ Household ☐ Neighborhood<br>☐ Others, specify_________________	
**Time of Bite**	____________	
**Site of Bite (body part/s)**	___________________	
**Number of Wounds**	_____ Brief description of wound type ______________________________	
**Bite provoked?**		
Yes	☐ Description of the circumstance ______________________________	
No	☐ ____	
**Nature of Bite**	☐ Minor ☐ Moderate ☐ Severe	
**Bite Category (WHO Classification)**	☐ I ☐ II ☐ III	

Treatment Received

**Wound washed?**		
No	☐	
Water	☐	
With soap	☐	
Antiseptic		
Alcohol	☐	
Iodine	☐	
Others (specify)	☐ ________________________	
**Time interval between bite and wound treatment**	________ minutes/hours/days	
**Application of local irritants (e.g. bleach, gasoline)**	☐ Yes, specify______________ ☐ No	
**Wound dressed/bandaged?**	☐ Yes ☐ No	
**Wound sutured?**	☐ Yes ☐ No	
**If traditional method, specify**	☐ Tandok ☐ Stone ☐ Horn ☐ Ritual<br>☐ Others, specify _____________________	
**Check if vaccine administered**		
Day 0	☐ Date (MM/DD/YYYY) ______________	*Patient follow up starts 14 days after this date*
Day 3	☐ Date (MM/DD/YYYY) ______________	*Update Wise-Monkey entry of patient*
Day 7	☐ Date (MM/DD/YYYY) ______________	*Update Wise-Monkey entry of patient*
Day 28/30	☐ Date (MM/DD/YYYY) ______________	*Update Wise-Monkey entry of patient*
**Treatment Type**	☐ IM ☐ ID	
**Antitetanus**	☐ Yes ☐ No	
**Immunoglobulin Type**	☐ ERIG ☐ HRIG ☐ No	
**Dose amount of immunoglobulin**	______ml or _____IU	
**If patient is dead, signs observed prior to death of the victim**	______________________________<br>______________________________	*PROMPT FOR INVESTIGATION*<br>*ALL DEATHS SHOULD BE INVESTIGATED IN THE FIELD*

IF PATIENT DID NOT GO FOR OR DID NOT CONTINUE TREATMENT:

**Reason/s why patient did not get treatment:**	______________________________<br>______________________________	*ASK ABOUT STATUS OF BITING ANIMAL. IF CASE IS SUSPICIOUS, INVESTIGATE AND FOLLOW UP ON PATIENT FOR TREATMENT*

FORM 2. Patient follow up (14 days after Day 0 of PEP)

ID of bite victim ____________________________ *ID assigned by Wise-Monkey*
Name of the victim ____
Status of the victim ☐ Alive ☐ Dead, date of death(MM/DD/YYYY) ______________

Species of biting animal ______________________
Breed of biting animal ______________________
Sex of biting animal ☐ Male ☐ Female
Age of biting animal _____ years
_____ months

If owned: *If not owned skip questions about the owner*
Name of owner ___________________________________
Address of owner
House name/number and street ____
Sitio ____
Barangay ____
Municipality/city ____

Pet Management ☐ Pet ☐ Confined ☐ Free/Stray ☐ Wild
Was biting animal available for 10-14 day observation ☐ Yes ☐ No

Rabies vaccination status of dog, if known
Unvaccinated ☐
Vaccinated within the last 12 months ☐
Vaccinated more than 12 months ago ☐

Status of Animal
Healthy ☐ *IF HEALTHY, END HERE. NO NEED FOR FIELD INVESTIGATION*
Sick ☐ *PROMPT FOR INVESTIGATION*
Died ☐ *PROMPT FOR INVESTIGATION. MUST DO OUTBREAK INVESTIGATION*
Killed ☐ *PROMPT FOR INVESTIGATION*
Not traceable ☐ *PROMPT FOR INVESTIGATION. IF DOG NOT FOUND, CONTINUE PEP OF PATIENT*
Unknown ☐ *PROMPT FOR INVESTIGATION*

FORM 3. Animal Bite Investigation Form - to be completed in the field (only when field investigation is necessary)

Investigated by: ______________________________________

Date of investigation: ______________________

ID of bite victim: ________________________ *ID assigned by Wise-Monkey*

ANIMAL PROFILE

If owned: *If not owned skip questions about the owner*

Name of owner _____________________________

Address of owner

House name/number and street _____________________________

Sitio _____________________________

Barangay _____________________________

Municipality/city _____________________________

Telephone number of owner __________________

Species __________________

Breed __________________

Sex ☐ Male ☐ Female

Age ____ years

____ months

Pet Management ☐ Pet ☐ Confined

☐ Free/Stray ☐ Wild

Status of Animal ☐ Sick ☐ Died ☐ Killed

☐ Not traceable ☐ Unknown

Cause of Death ☐ Euthanasia ☐ Accident ☐ Found dead

☐ Illness ☐ Others, specify______________

Date of Death ____________

Time of Death ____________

IF SICK, duration of illness *Skip question if no one can answer (because owner is unknown)*

From (MM/DD/YYYY) ____________

To (MM/DD/YYYY) ____________

Rabies vaccination status of dog, if known ☐ Unvaccinated ☐ Vaccinated within the last 12 months *Skip question if no one can answer (because owner is unknown)*

☐ Vaccinated more than 12 months ago

Vaccination History *Skip question if no one can answer (because owner is unknown)*

Rabies ☐ Date (MM/DD/YYYY) _______________

Other ____________________ ☐ Date (MM/DD/YYYY) _______________

Availability of biting animal for 10-14 day observation ☐ Yes ☐ No

Classification ☐ Proven rabid ☐ Suspect rabid ☐ Healthy *To be completed after lab test*

Postmortem/lab test report ☐ Yes ☐ No

Result of postmortem/lab test report ☐ Positive ☐ Negative

Number of dogs owned _________ *Skip question if no one can answer (because owner is unknown)*

Estimated number of dogs in the community _________

**Behavioral Changes**	**Check**	**Other Signs of Illness**	**Check**
None	☐	None	☐
Restlessness	☐	Diarrhea	☐
Apprehensive watchful look	☐	Vomiting	☐
Unprovoked aggressiveness	☐	Inappetence	☐
Aimless running	☐	Jaundice	☐
Deprived appetite	☐	Skin lesions	☐
Drooling saliva	☐	Lethargy	☐
Paralysis:	☐	Nasal/Ocular discharge	☐
Hindleg	☐	Convulsion/Seizures	☐
Jaw/Tongue	☐		

If these where observed even in an animal of unknown origin, COMPLETE THIS INFORMATION

Number of other persons bitten by the same animal (during the same week) _____ *Record their names* ________________________________

____

If no records for these victims, carry out patient follow up survey in field

Did biting animal bite other animals? ☐ Yes, date (MM/DD/YYYY) _______________ ☐ No

If Yes, record names of other animal owners: ______________________________

____

Carry out field investigation with the newly identified owner of other bitten animals

Was the animal bitten by a suspect rabid animal? ☐ Yes, date (MM/DD/YYYY) _______________ ☐ No

If yes, record owner of biting animal, if known: ____________________________________

If yes, carry out field investigation with the newly identified owner
